# Association Among BMI, Self-Esteem, and Nonsuicidal Self-Injury in Young Adults to Understand the Influence of Socioenvironmental Factors: Longitudinal Study

**DOI:** 10.2196/52928

**Published:** 2025-02-21

**Authors:** Yi Zhang, Ruixue Ying, Wan Lu, Xuemeng Liu, Keyan Hu, Qing Feng, Zixiang Yu, Zhen Wang, Fangting Lu, Yahu Miao, Nanzhen Ma, Fangbiao Tao, Tian Jiang, Qiu Zhang

**Affiliations:** 1 Department of Endocrinology, First Affiliated Hospital of Anhui Medical University, No 81 Meishan Road, Anhui, Hefei, 230032, China, 86 18356056506; 2 Department of Maternal, Child, and Adolescent Health, School of Public Health, Anhui Medical University, Hefei, China; 3 The First Affiliated Hospital and College of Clinical Medicine of Henan, University of Science and Technology, Luoyang, China; 4 Department of Pediatrics, The First Affiliated Hospital of Anhui Medical University, Hefei, China; 5 Hospital of Anhui Medical University, Hefei, China

**Keywords:** nonsuicidal self-injury, chronotype, BMI, self-esteem, body mass index, adolescent, young adult, teenager, social environmental factor, self-injury, sampling method, undergraduate, college student, linear regression, regression, regression model

## Abstract

**Background:**

Nonsuicidal self-injury (NSSI) is a major public health problem leading to psychological problems in adolescents and young adults, similar to disorders such as depression and anxiety.

**Objective:**

The aims of this study were to investigate (1) the interaction between BMI and socioenvironmental factors (including chronotype and mental health) that contribute to NSSI, and (2) whether self-esteem plays a mediating role in this association.

**Methods:**

From May to June 2022, the multistage cluster sampling method was used to sample college students in four grades, including freshmen and seniors. The baseline participants were followed up 6 months later, excluding those who did not qualify, and the participants included 1772 college students. Socioenvironmental factors (chronotype/mental health), self-esteem, and NSSI were measured using a questionnaire. Multivariate linear regression models and chi-square analysis were used to evaluate the linear relationship between BMI, socioenvironmental factors, and self-esteem and the NSSI status. We use a process approach (mediation-moderation analysis) to explore the complex relationships between these variables.

**Results:**

The mean age of the participants was 20.53 (SD 1.65) years at baseline. A significant association was revealed, suggesting that a high BMI (β=.056, 95% CI 0.008‐0.086, *P*=.018) was associated with a higher NSSI. There was also an interaction among BMI, socioenvironmental factors, and NSSI. Socioenvironmental factors played both moderating and mediating roles in the relationship between BMI and NSSI, whereas self-esteem only played a mediating role.

**Conclusions:**

Paying attention to factors such as overweight and obesity is important for early BMI control to identify other potential risk factors for NSSI and to evaluate how self-esteem can be improved considering multiple perspectives to improve the effect of BMI on NSSI in adolescents.

## Introduction

Nonsuicidal self-injury (NSSI) refers to physical harm, not the intention to commit suicide, and is not a socially recognized behavior [1]. NSSI, while being distinct from suicidal behavior, is a strong predictor of suicide and is also a significant mental health problem among adolescents worldwide [2]. NSSI is gradually increasing in the world; it seriously damages people’s physical and mental health and is a psychopathological behavior, which has aroused people’s attention and concern. The *DSM-5* identifies NSSI as a separate behavioral category and lists it as “an entry for further research” [3]. Adolescence and college years are important periods of psychological development and changes in life. In a meta-study, the reporting rate of NSSI was 22.5% (95% CI 17.2%‐28.9%) among adolescents [2]. The prevalence of NSSI ranged from 3.2% to 44.8% among nonclinical populations [4]. For clinical populations, the incidence of NSSI in the past 12 months ranged from 5% to 16.4% [4]. The harm of NSSI goes far beyond that, and after the emergence of NSSI in adolescence, it has maintained a certain “trajectory” into adulthood, including a continuous increase or fluctuation between the increase and decrease of NSSI, as well as an increase in psychobehavioral problems that largely affect people’s lives [5,6]. A systematic review and Bayesian meta-analysis concluded that in the entire developmental stage of adolescents, the NSSI frequency (but not the frequency of occurrence) in younger adolescents increased, that in middle adolescents remained stable, and that in older adolescents decreased [3]. On proceeding into the aftermath of the COVID-19 outbreak and trying to recover from its consequences, preventing NSSI and suicidal behaviors has continued to be a priority. In general, the current NSSI epidemic in China is serious [7], and it has become a major public health problem that severely endangers adolescents’ physical and mental health. Being an ongoing social health problem, NSSI is a major concern for mental health and it deserves our attention.

With such a high incidence rate of NSSI that has not been effectively controlled thus far, it is important to explore the potential factors of and prevention measures for NSSI, including what factors affect NSSI, how they interact with each other, and what needs to be done to protect those with high NSSI frequency. The specific factors that affect NSSI are as follows: (1) individual psychological factors, including self-abasement, impulsivity, early traumatic experience, self-esteem, and other characteristics; (2) external environmental factors, including negative life events (events that individuals feel unpleasant); (3) neurobiological factors, including the influence of the metabolism of substances such as 5-hydroxytryptamine, glutamate, and dopamine, which may also be related to genetics; (4) family factors, including family environment, family atmosphere, and family economic conditions; and (5) school factors, including school environment, teacher-student relationship, and classmate relationship, which may also have an impact on children and adolescents’ NSSI. The occurrence of NSSI behavior in adolescents is not the result of a single factor, but the result of the interaction of multiple factors. In addition, obesity has attracted considerable attention as a cause of many diseases, including physical diseases as well as psychological and behavioral problems [8]. Changes in BMI are correlated with suicide [9], and there is a significant association with suicide attempts in underweight and perceived overweight groups [10]. There is an association between psychological problems and NSSI [10,11]. A previous study demonstrated that while posttraumatic stress disorder leads to suicidal ideation, BMI plays a significant moderating role, indicating that the association between posttraumatic stress disorder and suicidal ideation is the strongest in individuals with high BMI values [11]. Another study has reported a correlation between obesity and NSSI and verified the correlation between BMI and major depressive disorder [12], but no precise research has explored the correlation between BMI and NSSI. NSSI is quite different from suicidal behavior in many aspects such as motivation, lethality, and manifestation; however, it remains a crucial risk factor for suicidal ideation and suicide attempts. This finding also suggests that psychological problems play an essential role in determining how BMI leads to NSSI.

Although some studies identified BMI as a risk factor for self-injury or suicide, other studies have not found positive results, which means that not everyone with an increased BMI necessarily shows NSSI behavior [13], as other factors may also be at play. To identify other possible psychological mechanisms that support this relationship, it is necessary to understand complex associations and mechanisms beyond the current one. In addition to the association between BMI and NSSI, it is significant to understand the mediators and moderators of BMI and NSSI for public health prevention and clinical practice. Research on borderline personality disorder has revealed that sleep deprivation, disrupted sleep, and prolonged sleep duration are associated with suicidal behavior or NSSI [14]. Sleep occupies a very important position in the circadian rhythm, especially the lack of sleep; the circadian rhythm refers to the 24-hour physiological and behavioral rhythm of individuals, which is divided into endogenous rhythm and exogenous rhythm. When the endogenous rhythm and exogenous rhythm are “mismatched,” circadian rhythms can be disrupted, which can lead to sleep disturbances [15]. Chronotype is a self-description of an individual’s circadian preference, reflecting the differences in their daily activity pattern and sleep-wake cycle; it can be generally divided into three types: morning, intermediate, and evening [16]. Moreover, the evening type has been reported to be strongly associated with suicidal behavior [17].

These studies provide evidence that NSSI moderates the relationship between BMI and socioenvironmental factors. A higher BMI has been associated with a lower risk of suicide in a large prospective study [18]; however, the mechanisms underlying this link require elucidation. Some studies have verified the correlation between self-esteem and NSSI [17,19]. However, little is known about how self-esteem moderates the relationship between BMI and NSSI. Socioenvironmental factors underlying these associations also remain unclear. Moreover, only limited research has been conducted on the relationship between BMI and socioenvironmental factors, and the outcome of NSSI during adolescence. A systematic review and meta-analysis reported that treatment of childhood obesity resulted in increased self-esteem after intervention [19]. The results of studies evaluating the self-esteem and NSSI suggest that although low self-esteem and self-injury are associated with both clinical and nonclinical populations, there are many factors influencing this relationship [20]. Therefore, based on a previous study [19], we hypothesize that there was an association among SE, BMI, and NSSI.

From a management and prevention perspective, these modifiable risk factors are crucial, and how they interact with each other requires further exploration. This study aimed to investigate the influencing factors and possible mechanisms of NSSI in college students and to observe the influence of some possible factors on BMI that could result in NSSI. Therefore, this study aimed to (1) examine the correlation among BMI, socioenvironmental factors, self-esteem, and NSSI and (2) determine whether socioenvironmental factors and self-esteem would play direct and indirect roles in the relationships between BMI and NSSI among Chinese college students.

## Methods

### Study Design and Study Setting for Recruitment

**Figure 1.**
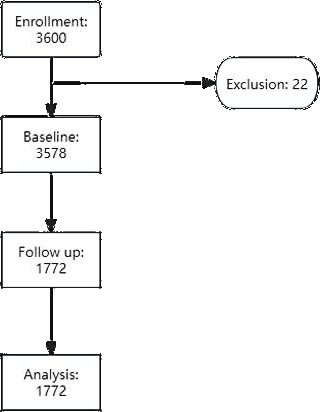
A flowchart for the enrollment and follow-up of participants.

This was a longitudinal study, and the research methods have been described in previous studies [21,22]. The study setting for recruitment was as follows: we first selected a university in a city in the Anhui Province, and then selected college students in four consecutive grades according to the cluster sampling method. A total of 3600 college students were initially recruited using an anonymous electronic questionnaire survey about their health at baseline. The cluster sampling method was used for the schools surveyed, and most of the students from each grade were included in the study. The survey was conducted from May to June 2022. Some participants were excluded from the study owing to unwillingness to answer the questionnaire, absence from class, high levels of missing data (questionnaires with missing values greater than 5% were eliminated), or false responses [23-25] at baseline. The baseline group was followed up with a questionnaire after 6 months. Finally, a total of 1772 participants were included in the follow-up survey (Figure 1).

### Inclusion Criteria

Participants were included if (1) informed consent from them and their guardians were obtained, (2) they were college students aged 15‐26 years, (3) they had no history of mental illness, and (4) they were attending the chosen school.

### Exclusion Criteria

Participants were excluded if (1) their informed consent was not obtained, (2) they were junior and high school students, (3) they failed to submit the questionnaire, or (4) they had congenital or acquired immunodeficiency.

### Exposure

#### Chronotype

The morningness-eveningness questionnaire is used to measure college students’ sleep habits. According to the previous analysis results, personal sleep habits is classified into three types: morning, intermediate, and evening [26]. The suggested demarcations were further categorized as follows: sleeping from 4 AM to 7 AM is definitely eveningness, sleeping from 8 AM to 11 AM is medium eveningness, sleeping from 12 PM to 5 PM is intermediate type, sleeping from 6 PM to 9 PM is medium morningness, and sleeping from 10 PM to 12 AM is definitely morningness.

### Self-Esteem

The self-esteem scale was first developed by Ronsenberg to evaluate an individual’s overall feelings of self-worth and self-acceptance [27]. It was later translated and revised into Chinese by Ji Fuyi and Yu Xin [28]. There were 10 items on the scale: 5 items on self-denial and 5 items on self-affirmation. A 4-point Likert scale was used, ranging from 1 indicating “very inconsistent” to 4 indicating “very consistent.” The 5 self-denial items were scored inversely. The higher the score, the higher the self-esteem.

### Outcome

#### NSSI

One of the most important question in the questionnaire was “Have you intentionally hurt yourself in the past 12 months, but not for suicide?” The questionnaire also listed several NSSI methods: hit yourself with a fist or palm, pulled your hair, hit a hard object with your head or fist, pinched or scratched yourself, bit yourself, cut yourself, and stabbed yourself. Those who had an NSSI were asked about their NSSI frequency. The participants owning up to 5 or more methods were considered to be prone to NSSI [29].

### Statistics Analysis

Conventional means and SDs were used for continuous variables. Univariate ANOVA was used to describe the association between independent and outcome variables. Frequency and percentage tests were used for categorical variables, and chi-square tests were used to explore the association between independent and outcome variables. The PROCESS method was used to conduct a mediation-moderation analysis to explore the relationships among BMI, chronotype, self-esteem, and NSSI [30]. The bootstrap method was used to resample 1000 samples, and the 95% CI was calculated. All data were analyzed using SPSS software (Windows version 23.0; IBM Corp).

### Ethical Considerations

The current study is designed and reported according to the STROBE (Strengthening the Reporting of Observational Studies in Epidemiology) checklist. The design and data collection procedures were approved by the Ethics Committee of the First Affiliated Hospital of Anhui Medical University (review number PJ 2024-06-06). Written informed consent was obtained from the parents or guardians of all the students.

## Results

### General Demographic Characteristics

**Figure 2.**
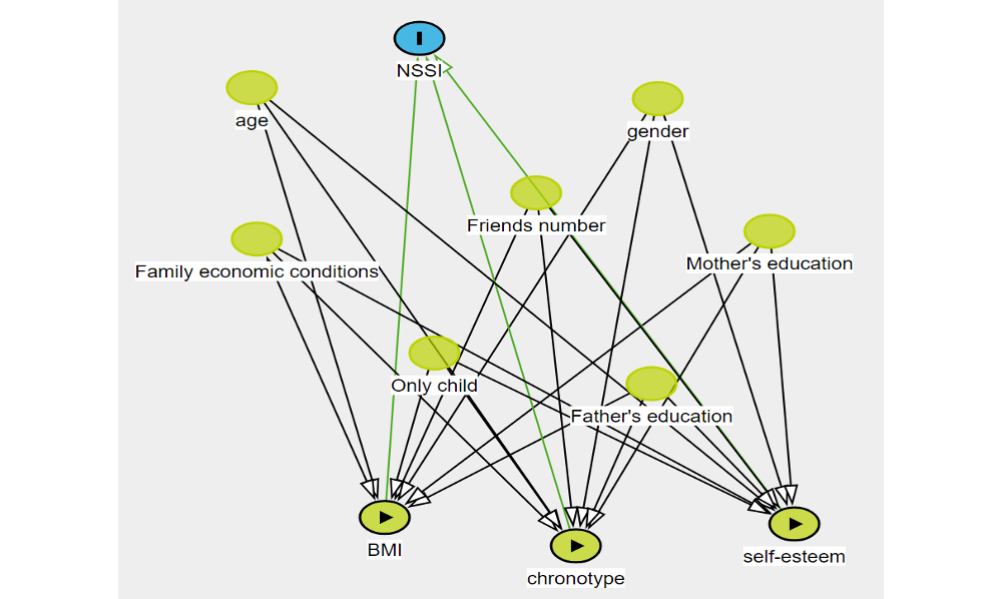
A directed acyclic graph showing the covariates. NSSI: nonsuicidal self-injury.

Table 1 presents the general distribution of the NSSI variables examined in this study considering the demographic characteristics, while Figure 2 is a directed acyclic graph showing the covariates. Among the 1772 participants included in the questionnaire analysis, 908 (51.1%) were male students; the mean age was 20.53 (SD 1.65) years at baseline; and 853 (48.1%) dwelled in rural areas, 548 (30.9%) in towns, and 371 (20.9%) in urban areas. The prevalence of NSSI was 5.6% (N=1772).

**Table 1.** The distribution of the nonsuicidal self-injury (NSSI) variables considering demographic characteristics.

Variables	Total, n (%)	Absence of NSSI, n (%)	Presence of NSSI, n (%)	*t* test*/*chi-square (*df*)	*P* value
Sex				0.46 (1)	.54
Male	908 (51.1)	854 (51.0)	54 (54.5)		
Female	864 (48.9)	819 (49.0)	45 (45.5)		
Residential areas				0.53 (2)	.76
Rural	788 (44.5)	744 (44.5)	44 (44.4)		
Town	401 (22.6)	376 (22.5)	25 (25.3)		
Urban	583 (32.9)	553 (33.1)	30 (30.3)		
Only child				0.29 (1)	.66
Yes	581 (32.8)	551 (32.9)	30 (30.3)		
No	1191 (67.2)	1122 (67.1)	69 (69.7)		
Father’s education				2.31 (5)	.81
Not known	55 (3.1)	4 (4.0)	51 (3.0)		
Below primary	153 (8.6)	7 (7.1)	146 (8.7)		
Primary	205 (11.6)	11 (11.1)	194 (11.6)		
Junior high	661 (37.3)	32 (32.3)	629 (37.6)		
High school or technical secondary school	379 (21.4)	24 (24.2)	355 (21.2)		
Junior college or above	319 (18.0)	21 (21.2)	298 (17.8)		
Mother’s education				3.14 (5)	.68
Not known	67 (3.8)	63 (3.8)	4 (4.0)		
Below primary	345 (19.5)	323 (19.3)	22 (22.2)		
Primary	316 (17.8)	299 (17.9)	17 (17.2)		
Junior high	535 (30.2)	504 (30.1)	31 (31.3)		
High school or technical secondary school	301 (17.0)	290 (17.3)	11 (11.1)		
Junior college or above	208 (11.7)	194 (11.6)	14 (14.1)		
Family economic conditions				3.15 (4)	.53
Bad	93 (5.2)	90 (5.4)	3 (3.0)		
Worse	428 (24.2)	402 (24.0)	26 (26.3)		
Medium	1142 (64.4)	1075 (64.3)	67 (67.7)		
Better	100 (5.6)	97 (5.8)	3 (3.0)		
Good	9 (0.5)	9 (0.5)	0 (0)		
Number of friends				4.92 (3)	.18
0	36 (2.0)	32 (1.9)	4 (4.0)		
1‐2	587 (33.1)	548 (32.8)	39 (39.4)		
3‐5	854 (48.2)	810 (48.4)	44 (44.4)		
6 or more	295 (16.6)	283 (16.9)	12 (12.1)		

### Association Between Independent Variables and Adolescent Psychological and Behavioral Problems

Table 2 presents the correlation between independent variables and depression symptoms through multilevel linear regression analyses. There was a significant relationship between different BMI and NSSI (β=.056); after controlling for covariates, these relationships were also significant. Furthermore, there was a significant relationship between self-esteem and NSSI (β=−.077); after controlling for covariates, this relationship was also significant. There was no significant correlation between chronotype and NSSI.

**Table 2.** Multilevel linear regression between independent variables and nonsuicidal self-injury (NSSI).

	NSSI						
	*R* ^2^	β	*t* test (*df*)	*P* value	*F* test (*df*)	95% CI	
BMI							
Model 1^a^	0.003	.056	2.359 (1)	.02	5.566 (1)	0.008 to 0.086	
Model 2^b^	0.008	.054	2.191 (9)	.03	1.551 (9)	0.005 to 0.085	
Chronotype							
Model 1	0.00	.022	0.938 (1)	.99	0.88 (1)	−0.033 to 0.094	
Model 2	0.006	.025	1.055 (9)	.29	1.139 (9)	−0.03 to 0.099	
Self-esteem							
Model 1	0.006	−.077	−3.234 (1)	<.001	10.461 (1)	−0.136 to −0.033	
Model 2	0.01	−.078	−2.896 (9)	.004	1.951 (9)	−0.130 to −0.025	

a Model 1: crude model.

b Model 2: controlled for parent educational level, gender, economic level, whether only child, number of friends, residential area, and age.

### Impact of the Mediation Analysis Among BMI, Chronotype, and Self-Esteem on Adolescent NSSI

Mediation-moderation analyses were performed for BMI, self-esteem, chronotype, and NSSI scores. The results are listed in Tables3  4. Among NSSI, findings have suggested the following: a positive association between BMI exposure and NSSI, chronotype was associated with NSSI, and chronotype played a moderating role in the increased risk of NSSI induced by BMI mediated by self-esteem.

**Table 3.** Testing the mediation-moderation effects of BMI^a^ and chronotype on the nonsuicidal self-injury (NSSI)^b^ among college students.

Variables	Self-esteem			NSSI		
	ß	*t* test (*df*)	*P* value	ß	*t* test (*df*)	*P* value
BMI	.0775	−0.4799 (3)	.54	.2262	2.5466 (5)	.01
Chronotype	−.0387	0.6206 (3)	.63	.8102	2.9399 (5)	.003
BMI* chronotype^c^	−.0005	−0.0869 (3)	.93	−.0127	−2.1149 (5)	.04
Self-esteem^d^	N/A^e^	N/A	N/A	.1613	1.3426 (5)	.18
Self-esteem* chronotype	N/A	N/A	N/A	−.0167	−2.0839 (5)	.04
*R* ^2^	0.0065	N/A	N/A	.0140	N/A	N/A
*F* test (*df*)	3.8342	N/A	N/A	5.0212	N/A	N/A

a BMI: independent variables.

b NSSI: dependent variables.

c chronotype: moderated variables.

d Self-esteem: mediate variables.

e N/A: not applicable.

**Table 4.** Bootstrap method showing the conditional direct and indirect effects between self-esteem, chronotype, and nonsuicidal self-injury (NSSI).

	Nonsuicidal self-injury		
	Effect of chronotype	Self-esteem	Lower limit to upper limit
Direct effect	0.0818	0.0270	0.0288 to 0.1347
Predictor	0.0409	0.0199	0.0020 to 0.0799
Moderator	0.0001	0.024	−0.0556 to 0.0558
Indirect effect	11.3542	0.0013	−0.0011 to 0.0053
Predictor	14.5643	0.0038	0.0006 to 0.008
Mediator	17.7744	0.004	−0.0002 to 0.0179

### Impact of Mediation Analysis Among BMI, Mental Health, and Self-Esteem on Adolescent NSSI

Mediation-moderation analyses were performed for BMI, self-esteem, mental health, and NSSI. The results are presented in Tables5  6. In NSSI, results have suggested a positive association between BMI exposure and NSSI, where depression was associated with NSSI, and chronotype played a moderating role in the increased risk of NSSI induced by BMI and mediated by self-esteem (Tables5  7). Similar results were obtained for stress and NSSI (Tables6  8).

**Table 5.** Testing the mediation-moderation effects of BMI^a^ and anxiety on the nonsuicidal self-injury (NSSI)^b^ among college students.

Variables	Self-esteem			NSSI		
	ß	*t* test (*df*)	*P* value	ß	*t* test (*df*)	*P* value
BMI	−.0099	−0.1054 (3)	.91	−.2475	−2.2641 (5)	.02
Anxiety	−.1302	−2.5744 (3)	.01	.0233	0.222 (5)	.82
BMI* anxiety^c^	−.0007	−0.2931 (3)	.77	.0070	2.6944 (5)	.007
Self-esteem^d^	N/A^e^	N/A	N/A	.0233	1.2468 (5)	.21
Self-esteem* anxiety	N/A	N/A	N/A	−.0051	−1.8281 (5)	.07
*R* ^2^	.1014	N/A	N/A	.0180	N/A	N/A
*F* test (*df*)	66.5210	N/A	N/A	6.4595	N/A	N/A

a Independent variables: BMI.

b Dependent variables: NSSI.

c Moderated variables: depression.

d Mediate variables: self-esteem.

e N/A: not applicable.

**Table 6.** Bootstrap method showing the conditional direct and indirect effects between self-esteem, anxiety, and nonsuicidal self-injury (NSSI).

	Nonsuicidal self-injury		
	Effect of anxiety	Self-esteem	Lower limit to upper limit
Direct effect	−0.0242	0.0343	−0.0914 to 0.0430
Predictor	0.02390	0.0212	−0.0177 to 0.0655
Moderator	0.0719	0.0238	0.0252 to 0.1186
Indirect effect	0.0001	0.0018	−0.0026 to 0.0051
Predictor	0.0016	0.0017	−0.0006 to 0.0063
Mediator	0.0044	0.0028	0.0001 to 0.0121

**Table 7.** Bootstrap method showing the conditional direct and indirect effects self-esteem, depression, and nonsuicidal self-injury (NSSI).

	Nonsuicidal self-injury		
	Effect of depression	Self-esteem	Lower limit to upper limit
Direct effect	−0.0273	0.0325	−0.0911 to 0.0364
Predictor	0.0328	0.0201	−0.0066 to 0.0722
Moderator	0.0929	0.0273	0.0393 to 0.1465
Indirect effect	0.0005	0.0020	−0.0016 to 0.0081
Predictor	0.0022	0.0017	0.00 to 0.0078
Mediator	0.0044	0.0035	−0.000 to 0.0148

**Table 8.** Testing the mediation-moderation effects of BMI^a^ and depression on the nonsuicidal self-injury (NSSI)^b^ among college students.

Variables	Self-esteem			NSSI		
	ß	*t* test (*df*)	*P* value	ß	*t* test (*df*)	*P* value
BMI	.0549	0.7267	.47	−.1616	−1.8212	.07
Depression^c^	−.0766	−2.3582	.02	−.0008	−0.0106	.99
BMI* depression	−.0016	−1.1464	.25	.0040	2.3457	.02
Self-esteem^d^	N/A^e^	N/A	N/A	.0516	0.5076	.61
Self-esteem* depression	N/A	N/A	N/A	−.0026	−1.2582	.21
*R* ^2^	.1236	N/A	N/A	.0130	N/A	N/A
*F* test (*df*)	83.1489	N/A	N/A	4.6602	N/A	N/A

a Independent variables: BMI.

b Dependent variables: NSSI.

c Moderated variables: depression.

d Mediate variables: self-esteem.

e N/A: not applicable.

## Discussion

### Principal Findings

In our study, the prevalence of NSSI in a sample of college students was 5.6%. Here, we examined the combination of chronotype and mental health as well as the interaction effect among BMI, self-esteem, and NSSI. BMI was positively correlated with the development of NSSI during adolescence, that is, the higher the BMI, the higher was the incidence of NSSI. Consistent with our hypothesis, our study provided evidence that self-esteem mediates the relationship between BMI and NSSI; this relationship was also moderated by chronotype. The relationship among BMI, self-esteem, and NSSI is complex and may involve a moderating effect of chronotype. Therefore, this study developed and evaluated a mediation-moderation model to elucidate the role of multilevel factors, including BMI, self-esteem, and chronotype, in relation to NSSI [31].

### Comparison With Other Studies

With the change in social living environment, adolescents are facing increasing pressure and challenges, and adolescent NSSI has become a problem that cannot be ignored. NSSI is a common mental health problem among adolescents [32]. In a college student–related survey, approximately 1 in 5 (20.4%) students reported lifelong NSSI, and the frequency of persistent NSSI was estimated at 56.4%, with 15.6% of students reporting high-frequency repeat patterns (≥5 times per year) [33]. This high prevalence is worth considering, and NSSI is not only associated with suicidal behavior but is also a specific risk factor for suicide. In addition to the burden on individuals and families, including NSSI and self-harm with suicidal intent, the increased incidence of NSSI requires significant health care and economic costs and can lead to death in severe cases, further emphasizing the need to study why people should be concerned about NSSI [34]. Therefore, it is necessary to pay attention to the factors that affect NSSI; some factors have been explored [35-38], such as adverse life events, stressful life events, negative coping styles, problematic internet use, sleep disorders, traumatic experiences, problematic parent-child relationships, and mental health issues. In the same way that obesity causes physical diseases (eg, cardiovascular disease and diabetes), obesity has a significant triggering effect on the number of times of suicide attempts [39]. However, a population-based telephone survey of US adults conducted by the Centers for Disease Control and Prevention found that traditional suicide risk factors do not show any correlation with BMI; therefore, there is unlikely to be any relationship between BMI and a lower risk of suicide [18]. That said, in some cases, it is not entirely the case that a higher BMI is associated with a greater number of risk factors. Further research into the relationship between BMI and suicide may lead to new modifiable risk factors that can result in or prevent this key cause of death [18]. This also encourages us to look beyond the known causes of suicidal behavior, as there are other factors at play in psychological research.

In the aforementioned research, individual factors are very important, and self-esteem is an important individual factor [20]. Such studies might shed light on how obesity interacts with chronotype factors and can affect self-esteem in people with NSSI [40]. Regarding the association between obesity and suicide-related variables, 3 out of 5 studies investigating these suicide attempts showed a positive association between these attempts and obesity, and this relationship may be related to the reduction in self-worth and self-esteem caused by obesity-related stigma, suggesting that self-esteem plays a key role in BMI-induced suicide attempts [20, 41]; the results suggest that although low self-esteem and NSSI are correlated in both clinical and nonclinical populations, many factors influencing this relationship still exist [20], which needs to be confirmed by further research. According to the developmental psychopathological model, individuals’ negative understanding and evaluation of themselves will lead to the emergence of NSSI, so low self-esteem is an important variable that triggers NSSI. Therefore, another study demonstrated the mediating role of self-esteem in the relationship between BMI and depression or suicidality, as well as the moderating role of sex in the mediated pathways [20], which also provides an effective theoretical basis for our research. It is noteworthy that self-esteem is a crucial factor given its significance in depression and NSSI. This study, thus, converges with the existing literature and extends it. Moreover, we noticed that chronotype patterns could moderate these relationships, and sleep deprivation itself could influence an increase in psychobehavioral problems [42]. Another review concluded that people with bipolar disorder are characterized by extreme mood swings, high rates of suicide, sleep problems, and dysfunction in psychological characteristics, such as self-esteem (a feeling ranked below depression and above mania), which further provides a theoretical basis for understanding the relationship between sleep, self-esteem, and metabolism [43]. The current findings highlight the importance of assessing self-esteem in mediating BMI and NSSI, perhaps targeting self-esteem to mitigate self-esteem–related mental health problems (eg, NSSI and suicide). We also considered the effect of the moderator mental health on BMI and NSSI. The stigma associated with being overweight has increased in recent decades, which may have exacerbated the harmful effects of being overweight on mental health [44], thereby further elevating the occurrence of NSSI.

Another study explored the relationship between adolescents’ BMI and mental health issues, including social phobia, depression, suicidal tendencies, and low self-esteem, and the mediating role of sex in participation in bullying [45]. Therefore, this provides a theoretical basis for our study that BMI is closely related to self-esteem, mental health, and suicidal behavior, and the correlation among the variables is also affected by other variables. According to the US Health and Retirement Study, baseline BMI, BMI transition, and BMI trajectory play significant moderating roles in the positive association between adverse childhood experience with new-onset all-cause dementia and Alzheimer disease [46]. The possible mechanisms include increased insulin and glucagon levels in the plasma and cerebrospinal fluid [47]. Additionally, our findings highlight the need for a multidisciplinary, collaborative, and integrated approach to optimize patient care. Different mechanisms have been identified, including gut-brain mechanisms, inflammatory responses (changes in the neutrophil-lymphocyte ratio, monocyte-lymphocyte ratio, and platelet-lymphocyte ratio), and changes in cytokine levels (interferon-γ, interleukin-1β, interleukin-6, interleukin-8, monocyte chemoattractant protein-1, tumor necrosis factor-α, and transforming growth factor-β1) [48].

### Clinical Practices

Overweight and obese adolescents often experience prejudice due to the surrounding environment, which causes them to have low self-esteem, depression, and other negative emotions; engage in bad behaviors; cause self-injury; and harbor suicidal thoughts. As sociality is an essential attribute of adolescents, overweight and obese adolescents should communicate with others and obtain support, thereby effectively reducing the occurrence of NSSI. By further exploring the two variables of chronotype and self-esteem, this study aimed to explore the sociological pathway of BMI leading to NSSI.

### Strengths and Limitations

The strengths of this study are its longitudinal design and large number of participants. The purpose of mediating is to strengthen the quasi-causal inference about the mechanism by which independent variables influence outcomes, whereas the purpose of moderating is to examine variables that influence the strength and direction of the predictor-outcome relationship. The use of moderated mediation could help identify the interdependence between the BMI and chronotypes. We also explored the role of the processes between early BMI and NSSI later in life.

However, this study has some limitations. First, although this was a follow-up study, only one follow-up survey was conducted, and changes in BMI were not evaluated during this process. These data are self-reported survey data, which can inevitably produce potential recall bias; therefore, we will further correct for the bias caused by recall bias in the future. Second, the study examined results in only one city; it is not clear how representative this sample is. Therefore, follow-up surveys are needed with samples from different regions and cultures across the country. Third, although this study mainly focuses on the relationship between BMI and NSSI under the influence of different factors, biological indicators will be included for further discussion in the future. Finally, we must consider critical attitudes toward the potential mechanism. As only some variables were evaluated in this study, additional variables with psychosocial factors should be considered in the future [48].

### Conclusion

In this study, there was a combination effect of BMI, self-esteem, and NSSI, as well as an interaction effect among BMI, self-esteem, and NSSI. NSSI prevention programs should focus on evidence-based approaches relevant to public health and address them in conjunction with relevant influencing factors, including a wide range of interventions such as self-help programs, education, policy change, and legislation. Stronger inferences about the role of BMI in the etiology of adolescent NSSI can be made by exploring sex and ameliorating unfavorable mental health in prospective cohort studies. Future studies should expand upon our small subset of measured variables to paint a broader picture of adolescent NSSI in response to early-life social, psychological, and environmental stimuli.

## Abbreviations

NSSI: Nonsuicidal self-injury
